# Lipoprotein(a) accelerated the progression of atherosclerosis in patients with end-stage renal disease

**DOI:** 10.1186/s12882-018-0986-2

**Published:** 2018-08-02

**Authors:** Kun Ling Ma, Tie Kai Gong, Ze Bo Hu, Yang Zhang, Gui Hua Wang, Liang Liu, Pei Pei Chen, Jian Lu, Chen Chen Lu, Bi Cheng Liu

**Affiliations:** 1 Institute of Nephrology, Zhong Da Hospital, Medical School of Southeast University, NO. 87, Ding Jia Qiao Road, Nang Jing City, 210009 Jiang Su Province China; 2 Renal Department, Danyang First People’s Hospital, Jiang Su Province, China

**Keywords:** Lipoprotein (a), Atherosclerosis, LDLr, CXCL16, End-stage renal disease

## Abstract

**Background:**

Increased plasma level of lipoprotein(a) (Lpa) is a risk factor of cardiovascular diseases. This study aimed to explore the role of Lpa in the progression of atherosclerosis in patients with end-stage renal disease (ESRD) and to investigate whether its potential mechanism is mediated by CXC chemokine ligand 16 (CXCL16) and low-density lipoprotein receptor (LDLr).

**Methods:**

This is a retrospective clinical study. From January 2015 to April 2016, forty-six ESRD patients from Danyang First People’s Hospital were investigated. The patients were grouped according to their plasma Lpa levels: control group (Lpa < 300 mg/l, *n* = 23) and high Lpa group (Lpa ≥ 300 mg/l, *n* = 23). ESRD Patients with acute infective diseases, cancer, and/or chronic active hepatitis were excluded. Biochemical indexes and lipid profiles of the patients were measured. Surgically removed tissues from the radial arteries of ESRD patients receiving arteriovenostomy were used for the preliminary evaluation of atherosclerosis. Haematoxylin-eosin (HE) and filipin staining were used to observe foam cell formation. Protein expression levels of Lpa, CXCL16, and LDLr were detected by immunohistochemistry staining and immunofluorescent staining.

**Results:**

There was more foam cell formation and cholesterol accumulation in the radial arteries of the high Lpa group than in those of the control group. Furthermore, the expression levels of Lpa, CXCL16, and LDLr were significantly increased in the radial arteries of the high Lpa group. Correlation analyses showed that the protein expression levels of Lpa (*r* = 0.72, *P* < 0.01), LDLr (*r* = 0.54, *P* < 0.01), and CXCL16 (*r* = 0.6, *P* < 0.01) in the radial arteries of ESRD patients were positively correlated with the plasma Lpa levels. Further analyses showed that the co-expression of Lpa with LDLr or CXCL16 was increased in the high Lpa group.

**Conclusions:**

High plasma Lpa levels accelerated the progression of atherosclerosis in ESRD through inducing Lpa accumulation in the arteries, which was associated with LDLr and CXCL16. These two lipoproteins could both be major lipoprotein components that regulate the entry of Lpa into arterial cells.

## Background

Cardiovascular diseases (CVDs), which are mainly caused by atherosclerosis, are the leading cause of death in end-stage renal disease (ESRD) patients [1]. The incidence of atherosclerosis in ESRD patients is significantly higher than that in the general population [2]. It was found that genetically increased levels of lipoprotein(a) (Lpa) were associated with an increased risk of coronary disease [3]. In the Atherosclerosis Risk in Communities Study, Virani et al. illustrated that plasma Lpa levels were positively associated with CVD events [4]. Momiyama et al. demonstrated that plasma Lpa levels were positively correlated with atherosclerosis in the abdominal aorta and coronary artery [5]. However, the potential mechanisms underlying Lpa-induced atherosclerosis progression remain unclear.

Lpa contains two different polypeptides, one of which has been proven to correspond to the B-100 protein in LDL [6]. Romagnuolo et al. demonstrated that the low-density lipoprotein receptor (LDLr) mediated Lpa internalization in HepG2 cells by identifying the analogous structure of LDL [7]. Membrane-bound CXC chemokine ligand 16 (CXCL16) has been reported as a scavenger receptor that internalizes oxidized LDL [8] and might be involved in the progression of atherosclerosis [9].

Therefore, this study aimed to investigate the role of Lpa in the progression of atherosclerosis and to explore the association of Lpa deposition with LDLr and CXCL16 expression in radial arteries of ESRD patients.

## Methods

### Study design

This is a retrospective clinical study. From January 2015 to April 2016, forty-six ESRD patients from Danyang First People’s Hospital were investigated. The patients were grouped according to their plasma Lpa levels: control group (Lpa < 300 mg/l, *n* = 23) and high Lpa group (Lpa ≥ 300 mg/l, *n* = 23). ESRD Patients with acute infective diseases, cancer, and/or chronic active hepatitis were excluded.

### Tissue specimens

Excised radial artery tissues from ESRD patients were obtained during arteriovenous fistula surgery. After being washed with phosphate buffered saline (PBS), the tissues were immediately immersed in 4% paraformaldehyde for 24 h. Then, the tissues were dehydrated and embedded in paraffin. Paraffin sections with a thickness of 4 μm were used for subsequent experiments.

### Clinical biochemical assays

Blood samples from ESRD patients were obtained for the clinical biochemical assays. The plasma levels of Lpa, total protein (TP), albumin (ALB), triglycerides (TGs), total cholesterol (TC), high-density lipoprotein (HDL), low-density lipoprotein (LDL), apolipoprotein A1 (Apo A1), Apo B, calcium (Ca), phosphate (P), and intact parathyroid hormone (iPTH) were detected by a Coulter AU580 automatic biochemical analyzer (Beckman, USA). Red blood cells (RBCs) and haemoglobin (Hb) levels were measured by an automated haematology analyzer (Sysmex, Japan). The plasma levels of C-reactive protein (CRP) were detected by an automated protein analyzer (Siemens, Germany).

### HE staining

The sections were dewaxed and hydrated. The sections were then stained with Harris haematoxylin solution for 15 min at room temperature. After differentiation with 1% acid alcohol for 1 min, the sections were incubated with 1% eosin for 5 min.

### Filipin staining

The sections were dewaxed and hydrated. The sections were then incubated with a Filipin working solution (50 μg/ml) for 30 min at room temperature. The samples were observed by fluorescence microscopy. Five microscopic fields of each section (*n* = 5) were chosen randomly for fluorescence intensity evaluation by ImageJ software.

### Immunohistochemical staining

After deparaffinization, the sections were immersed in 2% EDTA solution and heated to boiling for antigen retrieval; next, the sections were blocked with 3% hydrogen peroxide for 10 min at room temperature. The slides were then incubated with primary antibodies for Lpa (Novus, USA), LDLr (Santa Cruz, USA) or CXCL16 (R&D, USA) at 4 °C overnight. After reaction with biotin-labelled secondary antibodies, the sections were incubated with diaminobenzidine to detect the positive signal with a light microscopy. Five patients from each group were randomly selected for statistical analyses. Five random microscopic fields of each section (*n* = 5) were chosen for optical density quantitation by Image-Pro Plus software.

### Immunofluorescent staining

After deparaffinization and antigen retrieval, the slides were treated with 0.25% Triton-100 for 15 min and blocked with 5% bull plasma albumin for 30 min at room temperature. Then, the sections were incubated with Lpa antibody at 4 °C overnight and reacted with the fluorescent secondary antibody for 2 h at room temperature. For double immunofluorescent staining, the sections were incubated with primary antibodies for CXCL16, Lpa or LDLr at 4 °C overnight, followed by staining with secondary antibodies. All slides were observed with immunofluorescence laser scanning confocal microscopy. Five microscopic fields of each section (*n* = 5) were chosen randomly for fluorescence intensity evaluation by ImageJ software.

### Statistical analysis

The sample size was based on available data. No statistical power calculation was conducted prior to the study. Data were analysed with SPSS 23.0. Normally distributed data are presented as the means±SD, and independent-sample t tests were used for comparisons between two groups. Abnormally distributed data are presented as the median (P25, P75), and Mann-Whitney U tests were used to compare the differences between two groups. Spearman’s correlations and Pearson correlations were used for correlation analyses. Differences were considered statistically significant when *P* < 0.05.

## Results

### Basic data for the patients in the two groups

As shown in Table 1, there were no significant differences in age, body weight, BMI, RBC, Hb, TP, ALB, GPT, GOT, TC, TG, LDL, HDL, ApoA1, ApoB, Ca, P, or iPTH levels (*P* > 0.05) between the high Lpa group and the control group (Table 1).

**Table 1:** Basic clinical and biochemical data for the patients

Parameters	Control (*n* = 23)	Lpa group (*n* = 23)
Original disease distribution (n)		
Chronic glomerulonephritis	14	16
Diabetic nephropathy	5	5
Nephrotic syndrome	0	1
Obstructive nephropathy	1	1
Hypertension	1	0
Lupus nephropathy	1	0
Polycystic kidney	1	0
Previous cardiovascular disease/events(n)	5/1	5/3
Sex (n)		
Female	11	7
Male	12	16
Age(y)	55.4±12.2	51.7±13.4
Weight(kg)	61.0±12.1	61.8±10.4
BMI(kg/m^2^)	22.2±2.9	22.2±2.3
Scr (μmol/L)	665 (543,823.2)	858 (694.2,1216.1)
WC(cm)	76.5±7.9	78.5±7.6
CRP(mg/L)	5.1(1.6,8.0)	4.8(2.7,12.1)
ESR(ml/h)	52.0±34.1	67.6±32.9
Hb(g/L)	76.3±15.6	78.0±16.6
RBC(× 10^12^/L)	2.66±0.54	2.74±0.51
TP(g/L)	62.6±8.2	58.9±10.2
ALB(g/L)	37.5±6.6	31.8±7.2
ALT(U/L)	14.73±7.19	14.01±8.61
AST(U/L)	19.3±1.6	19.15±1.35
TG (mmol/L)	1.61(0.86,2.69)	1.21(0.90,1.59)
T-CHO(mmol/L)	3.90±0.65	4.01±0.59
LDL (mmol/L)	1.94(1.85,2.08)	2.16(1.9,2.36)
HDL (mmol/L)	1.03(0.73,1.21)	0.99(0.92,1.15)
ApoA (mmol/L)	1.13(0.97,1.2)	1.14(1.01,1.35)
ApoB (mmol/L)	0.73±0.2	0.83±0.22
Ca(mmol/L)	2.13±0.25	1.99±0.27
P(mmol/L)	1.79±0.61	1.93±0.56
Ca×P(mmol^2^/L^2^)	47.79±15.74	48.31±15.51
iPTH (ng/L)	301.2(148.1373.4	273.2(130.4553.6)

There was no difference compared every index in the inflamed group with that in the control, *P* > 0.05

### Lpa expression levels in radial arteries were positively associated with plasma Lpa levels

**Fig. 1:**
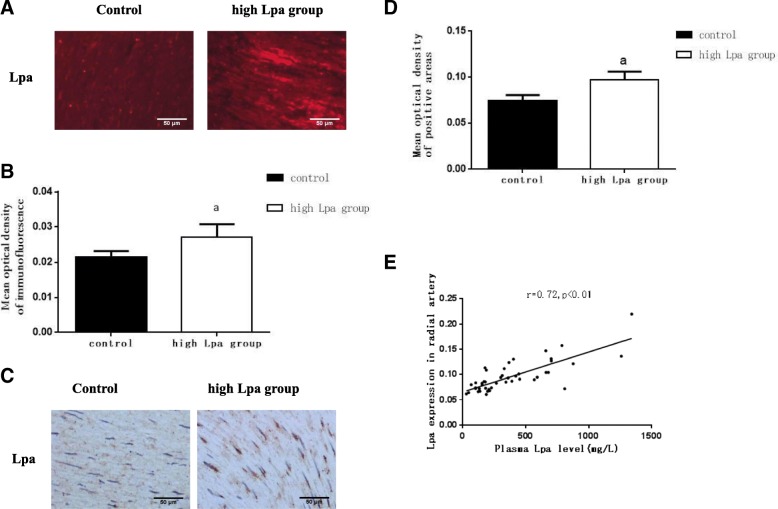
Lpa expression levels in the radial arteries were positively associated with the plasma Lpa levels. Protein expression levels of Lpa in the radial arteries were measured by (**a**) immunofluorescent staining (original magnification ×200) and (**c**) immunohistochemical staining (brown colour, original magnification × 200). The values of semiquantitative analyses of the positive areas were expressed as the mean ± SD of five patients from each group (*n* = 5). **b** 0.0284±0.0041 vs. 0.0221±0.0028. ^a^*P* < 0.05 for the high Lpa group compared with the control, respectively. **d** 0.1135±0.0268 vs. 0.0709±0.0085. ^a^*P* < 0.05 for the high Lpa group compared with the control, respectively. Correlation analysis of plasma CRP levels with Lpa expression levels (**e**)

As shown in Fig. 1, the Lpa expression levels in the radial arteries of the high Lpa group were significantly increased when compared with those in the control group (Fig. 1a-d). Spearman correlation analysis showed that the Lpa expression levels in the radial arteries of the ESRD patients were positively correlated with the plasma Lpa levels (Fig. 1e).

### Lpa accelerated foam cell formation and cholesterol deposition in the radial arteries of ESRD patients

**Fig. 2:**
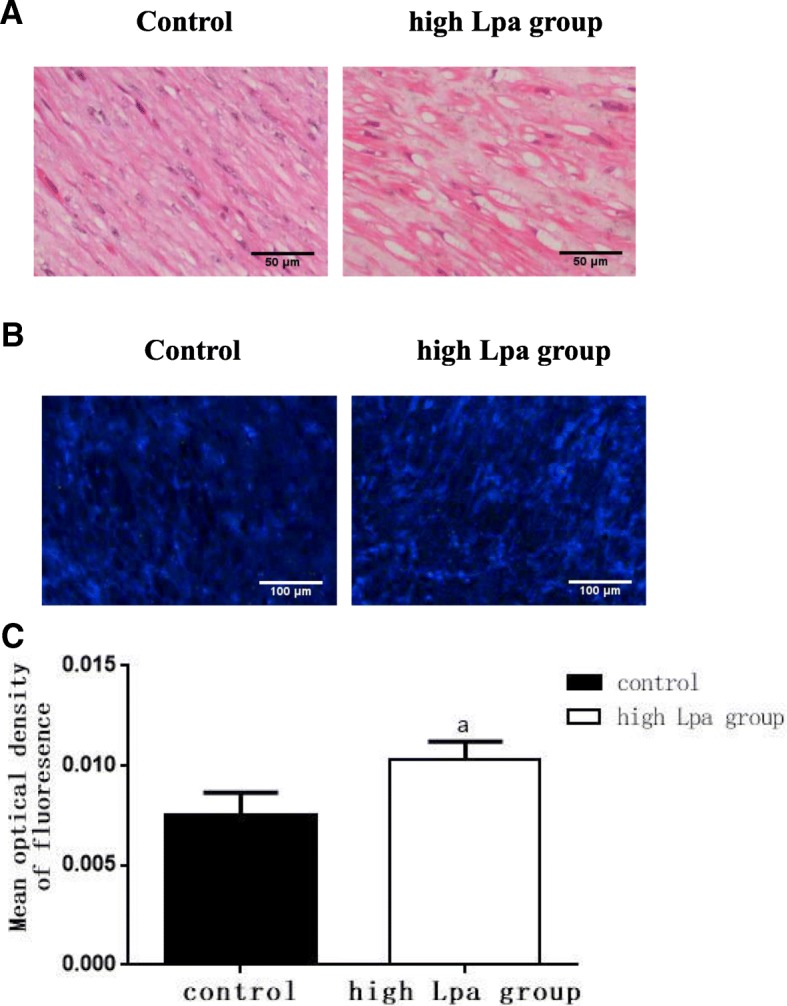
Lpa accelerated foam cell formation and cholesterol deposition in the radial arteries of ESRD patients. The lipid accumulation in the radial arteries was determined by (**a**) haematoxylin-eosin staining (original magnification × 400) and (**b**) filipin staining (original magnification × 200). The values of the semiquantitative analysis of the positive areas were expressed as the mean ± SD of five patients from each group (*n* = 5). **c** 0.0101±0.0010 vs. 0.0074±0.0012. ^a^*P* < 0.05 for the high Lpa group compared with the control, respectively

To evaluate the effects of high levels of plasma LPa on the progression of atherosclerosis, we observed foam cell formation by HE staining and cholesterol accumulation by filipin staining. Foam cell formation in the radial arteries was significantly higher in the high Lpa group than in the control group (Fig. 2a). Filipin staining showed that cholesterol accumulation in the radial arteries of the high Lpa group also increased (Fig. 2b-c).

### Increased CXCL16 and LDLr expression levels in the high Lpa group

**Fig. 3:**
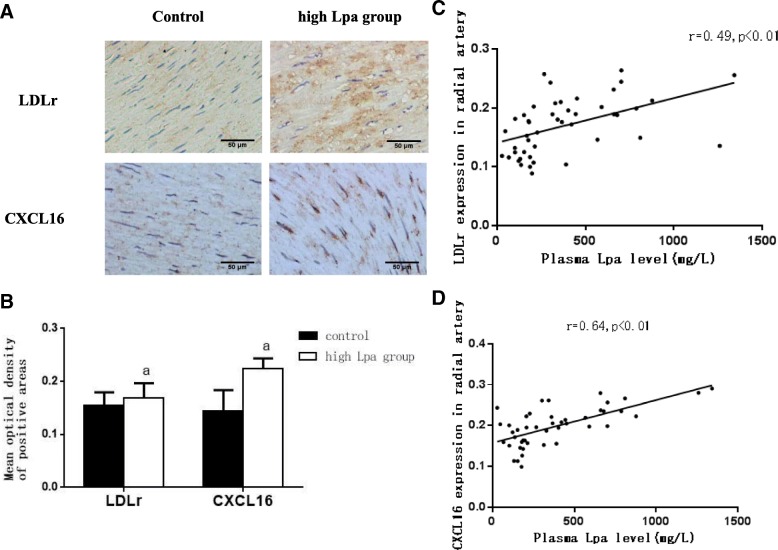
Increased CXCL16 and LDLr expression levels in the high Lpa group. The protein expression levels of LDLr and CXCL16 in the radial arteries were measured by (**a**) immunohistochemical staining (brown colour, original magnification × 200). The values of the semiquantitative analyses of the positive areas were expressed as the mean ± SD of five patients from each group (*n* = 5). **b** 0.1873±0.0389 vs. 0.1300±0.0270. ^a^*P* < 0.05. for the high Lpa group compared with the control, respectively. Correlation analysis of plasma Lpa levels with LDLr (**c**) and CXCL16 expression levels (**d**)

By using immunohistochemical staining, we found that the protein expression levels of CXCL16 and LDLr in the radial arteries of the high Lpa group were significantly higher than in the control group (Fig. 3a-b). Moreover, the expression levels of CXCL16 and LDLr in the radial arteries of the ESRD patients were positively correlated with their plasma Lpa levels (Fig. 3c-d).

### LDLr and CXCL16 modulated the transportation of plasma Lpa into the radial arteries

**Fig. 4:**
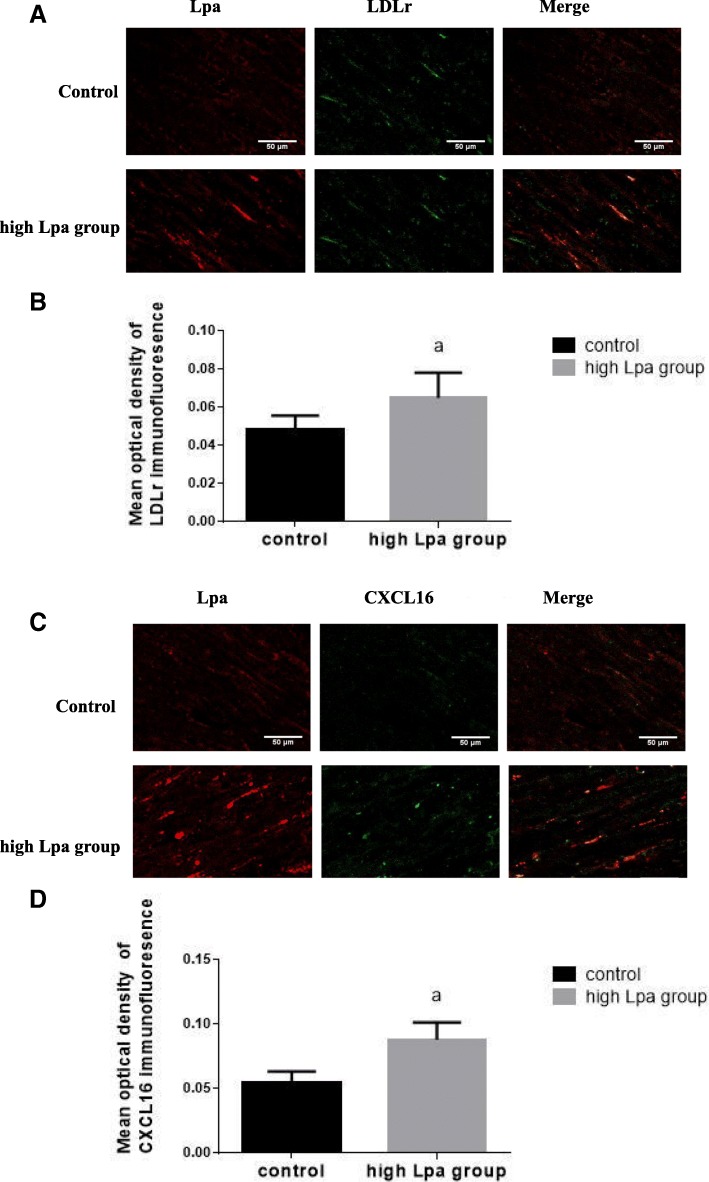
LDLr and CXCL16 modulated the transportation of plasma Lpa into the radial arteries. Lpa and LDLr were stained red and green, respectively; the colocalization of Lpa and LDLr was measured by (**a**) laser confocal microscopy (original magnification × 400). Lpa and CXCL16 were stained red and green, respectively; the colocalization of Lpa and CXCL16 was measured by **c** laser confocal microscopy (original magnification × 400). The values of semiquantitative analysis of the fluorescence intensity are expressed as the mean ± SD from five patients in each group (*n* = 5). **b** 0.0652±0.0131 vs. 0.0483±0.0075. ^a^*P* < 0.05. for the high Lpa group compared with the control, respectively. **d** 0.0881±0.0134 vs. 0.0546±0.0090. ^a^*P* < 0.01. for the high Lpa group compared with the control, respectively

Confocal microscopy observations showed that the co-expression of Lpa and LDLr was significantly increased in the high Lpa group, as well as the co-expression of Lpa and CXCL16 (Fig. 4a-d).

## Discussion

Many studies have proposed increased plasma Lpa levels as an independent risk factor for cardiovascular disease [10, 11]. Pedersen et al. found that increased Lpa expression levels accelerated uraemia-induced atherosclerosis, which might be mediated by the binding of oxidized phospholipids to Lpa [12]. Using a transgenic Watanabe heritable hyperlipidaemic rabbit model, Kitajima et al. reported that elevated plasma Lpa levels aggravated atherosclerosis in the coronary arteries and increased the incidence of myocardial infarction [13].

In this study, we observed that there was more Lpa deposition in the radial arteries of ESRD patients from the high Lpa group. Moreover, Filipin and HE staining showed that cholesterol accumulation and foam cell formation were significantly higher in the high Lpa group than in the control group. Our data suggest that high plasma Lpa levels might be the main cause of cholesterol accumulation and foam cell formation in the radial arteries of ESRD patients. As we know, hypercholesterolemia is a main risk factor for the progression of atherosclerosis. Lipoprotein (a), one of the components of plasma lipid profile, was shown similar effects with hypercholesterolemia on promoting systemic atherosclerosis. Therefore, high-Lpa induced radial atherosclerosis means that Lpa may contribute to the progression of cardiovascular disease in ESRD patients.

Clinical trials have demonstrated that proprotein convertase subtilisin/kexin type 9 inhibitors (PCSK9i) can significantly reduce LDL-cholesterol and Lpa levels [14]. Edmiston et al. found that the correlation between reduced LDL-cholesterol levels and Lpa levels in response to PCSK9i was moderate, and this result indicated that pathways beyond that of the LDLr were responsible for Lpa lowering [14]. Raal et al. reported that PCSK9 inhibitor-induced reductions in circulating Lpa levels partly depende on the LDLr-mediated uptake of Lpa [15]. The LDLr gene family member megalin/glycoprotein (gp) 330 was identified as the functional structure that binds to and mediates the cellular uptake of Lpa [16]. Yang et al. first reported that scavenger receptor class B type I bound to Lpa and facilitated Lpa-associated lipid uptake [17]. However, it is not clear whether CXCL16, which is also a scavenger receptor, can bind and internalize Lpa.

In this study, we found that LDLr and CXCL16 expression levels were increased in the high Lpa group, and the expression levels of these two proteins were positively correlated with the plasma Lpa levels. Confocal microscopy observations showed that Lpa colocalized with both LDLr and CXCL16 in the radial arteries of the ESRD patients. These data suggested that LDLr and CXCL16 might mediate the internalization of Lpa and thus contribute to lipid deposition in the radial arteries of ESRD patients.

## Limitations

The small sample size and tiny tissues from radial arteries in two groups may limit acquiring more evidence to evaluate atherosclerosis caused by Lpa. This is also a key limitation for this pilot study. More samples will be collected in our future work to improve the statistical power. In addition, as each patient had different specific conditions (age, sex, complications, treatments, serum levels of phosphate, calcium, and intact parathyroid hormone, etc.), the data could be not well-controlled.

## Conclusions

This study demonstrated that high plasma Lpa levels contributed to atherosclerosis in ESRD patients through increasing Lpa accumulation and foam cell formation in the radial arteries, and these actions were correlated with Lpa internalization mediated by LDLr and CXCL16.

## Abbreviations

ALB: Albumin
Apo A1: Apolipoprotein A1
Ca: Calcium
CRP: C-reactive protein
CVDs: Cardiovascular diseases
CXCL16: CXC chemokine ligand 16
ESRD: End-stage renal disease
Hb: Haemoglobin
HDL: High-density lipoprotein
iPTH: Intact parathyroid hormone
LDL: Low-density lipoprotein
LDLr: Low-density lipoprotein receptor
Lpa: Lipoprotein(a)
P: Phosphate
PCSK9i: Proprotein convertase subtilisin/kexin type 9 inhibitors
RBCs: Red blood cells
TC: Total cholesterol
TGs: Triglycerides
TP: Total protein
